# Leveraging Community Science to Measure Bee Body Size From Museum Specimens

**DOI:** 10.1002/ece3.71665

**Published:** 2025-06-21

**Authors:** Madeleine M. Ostwald, Colleen Smith, Julie Allen, Alec Buetow, A. Rosie Manner, Robert Guralnick, Carys Goldsmith, Katja C. Seltmann

**Affiliations:** ^1^ Cheadle Center for Biodiversity & Ecological Restoration University of California, Santa Barbara Santa Barbara California USA; ^2^ Department of Biological Sciences Virginia Tech Blacksburg Virginia USA; ^3^ Florida Museum of Natural History University of Florida Gainesville Florida USA

**Keywords:** body size, citizen science, digitization, insects, intertegular distance, natural history collections, pollinators

## Abstract

Community or volunteer participation in research has the potential to significantly help mobilize the wealth of biodiversity and functional ecological data housed in natural history collections. Many such projects recruit community scientists to transcribe specimen label data from images; a next step is to task community scientists with conducting straightforward morphological measurements (e.g., body size) from specimen images. We investigated whether community science could be an effective approach to generating significant body size datasets from specimen images generated by museum digitization initiatives. Using the community science platform Notes from Nature, we engaged community scientists in a specimen measurement task to estimate body size (i.e., intertegular distance) from images of bee specimens. Community scientists showed high engagement and completion of this task, with each user measuring 43.6 specimens on average and self‐reporting successful measurement of 98.0% of the images. Community scientist measurements were significantly larger than measurements conducted by trained researchers, though the average measurement error was only 2.3%. These results suggest that community science participation could be an effective approach for bee body size measurement, for descriptive studies or for research questions where this degree of expected error is deemed acceptable. For larger‐bodied organisms (e.g., vertebrates), where modest measurement errors represent a smaller proportion of body size, community science approaches may be particularly effective. Methods we present here may serve as a blueprint for future projects aimed at engaging the public in biodiversity and collections‐based research efforts.

## Graphical abstract

Community or volunteer participation in research has the potential to significantly help mobilize the wealth of biodiversity and functional ecological data housed in natural history collections. We tasked community scientists with measuring bee body size from images of museum specimens and assessed data quality. Community scientists showed high engagement and completion of this task; while their measurements were significantly larger than measurements conducted by trained researchers, the average measurement error was minimal (2.3%).

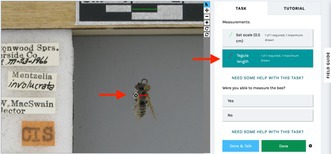

## 1 Introduction

Body size is a fundamental aspect of an organism's life history and ecology (Angilletta et al. 2004; Blackburn and Gaston 1994; Blueweiss et al. 1978; Brown et al. 2004; Peters 1983). Size influences social interactions (Pereira 1995; Reeve 1991), reproductive fitness (Bünger et al. 2005; Honek 1993; Kingsolver and Huey 2008), physiological processes (Brown et al. 2004; Gillooly et al. 2001; Klockmann et al. 2017), and the provisioning of ecosystem services (Guo et al. 2022; Jauker et al. 2016). Body size distributions can be affected by global change, with many taxonomic groups experiencing body size reductions in response to climate warming (Gardner et al. 2011; Kelemen and Rehan 2021; Merilä and Hendry 2014; Sheridan and Bickford 2011; Van Buskirk et al. 2010). Natural history collections offer valuable opportunities to document size shifts over time through specimens that span historic changes in climate and land use (Maclean et al. 2016; Theriot et al. 2023).

Accessing and mobilizing the wealth of biodiversity data housed in natural history collections has been a major focus of recent initiatives (Hedrick et al. 2020). Community science (also known as citizen science; i.e., the participation of volunteers in scientific data collection and analysis; Bonney et al. 2009; Vohland et al. 2021) has made important contributions to these efforts, in particular, through the crowd‐sourced transcription of museum collection data from images of specimen labels. Many such initiatives have accelerated the digitization and analysis of museum records, whereas offering avenues for public engagement in the scientific process (Ballard et al. 2017; Hedrick et al. 2020; Hill et al. 2012). Beyond the biodiversity occurrence and interaction data found in specimen labels, images of specimens themselves offer tantalizing opportunities for morphometric measurement (e.g., body size). Although community science has been an important tool for image annotation and classification tasks (Arandjelovic et al. 2024; Willi et al. 2019; Yost et al. 2018), continuous trait measurement represents a new challenge that has rarely been explored in a community science context. If successful, crowd‐sourcing trait measurements from museum specimen images could greatly extend our understanding of body size variation across time and geographic space. These measurements contribute to the extended specimen, that is, the multidimensional biodiversity and phenotypic datasets that extend beyond the physical specimen itself (Lendemer et al. 2020; Webster 2017). Measurement tasks also have the potential to offer new, engaging opportunities for public interaction with digitized biodiversity collections.

As the most significant animal pollinators (Ollerton et al. 2011), bees (Hymenoptera: Apoidea) represent an important target group for large‐scale studies of body size variation. Public interest in pollinator conservation (Nicholls et al. 2020) also positions bees as a promising group for community science engagement. Studies of museum specimens suggest that bee body size is sensitive to changes in climate and land use (Kelemen and Rehan 2021; Nooten and Rehan 2020). These changes in body size can have far‐reaching consequences for ecosystem functioning when they create functional mismatches with host plants (Miller‐Struttmann et al. 2015) or reduce pollination effectiveness (Jauker et al. 2016). Because size shifts may be species‐ or context‐specific, and because they may be confounded by other environmental changes (Herrera et al. 2023; Kelemen and Rehan 2021), our ability to understand the causes and consequences of body size change will depend on the generation of comprehensive datasets from historic and contemporary specimens.

We investigated community science data as an approach for generating large, robust body size datasets from museum specimens. Data quality assessments are an essential validation step for understanding the reliability and utility of community science data collection protocols, and for guiding the deployment of these projects and the use of their data (Balázs et al. 2021; Downs et al. 2021; Lukyanenko et al. 2016; Wiggins et al. 2011). We assessed measurement data quality by comparing community scientist measurements of bee body size to measurements conducted by trained researchers. We quantified body size as the distance between the wing bases or tegulae (i.e., intertegular distance, ITD), a common metric for intraspecific body size estimation in bees (Cane 1987; Kendall et al. 2019; Ostwald et al. 2024) that can be measured accurately from dorsal specimen images. We deployed these projects through the web‐based platform Notes from Nature, a tool developed by biodiversity scientists and community science experts to facilitate new approaches to specimen digitization (Hill et al. 2012). We use these results to better understand the utility of this approach for expanding our understanding of bee trait evolution and to provide best practice approaches for capturing the highest quality data and facilitating public interactions with rapidly digitizing biological collections.

## 2 Methods

### 2.1 Specimens and Imaging

To facilitate community scientist ITD measurements, we imaged 479 metallic green sweat bees (Halictidae:Halictinae) from the University of California, Santa Barbara Invertebrate Zoology Collection housed by the Cheadle Center for Biodiversity and Ecological Restoration. We chose these bees as our focal group because the bright color of the integument and the lack of dense hairs obscuring the tegulae reduces ambiguity in ITD measurement. These specimens were collected primarily in Santa Barbara County, CA, and included the following taxa: (Hymenoptera:Halictidae): *Agapostemon subtilior* Vachal, 1903 (*N* = 420), *Agapostemon melliventris* Cresson, 1874 (*N* = 1), *Agapostemon* sp. Smith, 1853 (*N* = 12), *Augochlorella pomoniella* Cockerell, 1915 (*N* = 40), *Augochlorella aurata* Smith, 1853 (*N* = 2), and *Augochloropsis metallica* Fabricius, 1793 (*N* = 1). Full specimen information is available at https://doi.org/10.5281/zenodo.14927710. All specimens were imaged in dorsal view using a Canon EOS 6D Mark II camera fitted with a Canon RF 100 Macro USM AF/MF lens. To calibrate measurements, we included a ruler in all images that was positioned level with the surface of the bees' thorax (i.e., the measurement surface).

### 2.2 Community Science Measurements

We obtained community scientist‐sourced body size measurements by uploading all specimen images to the Zooniverse platform Notes from Nature and creating an “expedition” that engages users to participate in a set of tasks related to those images. Notes from Nature utilizes services made available by Zooniverse to coordinate community science activities related to natural history collections. These services include tools for zooming, panning, and annotating images, as well as a forum for participants to discuss their experiences and interact with scientists guiding the expedition. When creating an expedition, scientists curate a set of images, set up key tasks, develop tutorials and help guides, and explain the value of the work for scientists.

**FIGURE 1:**
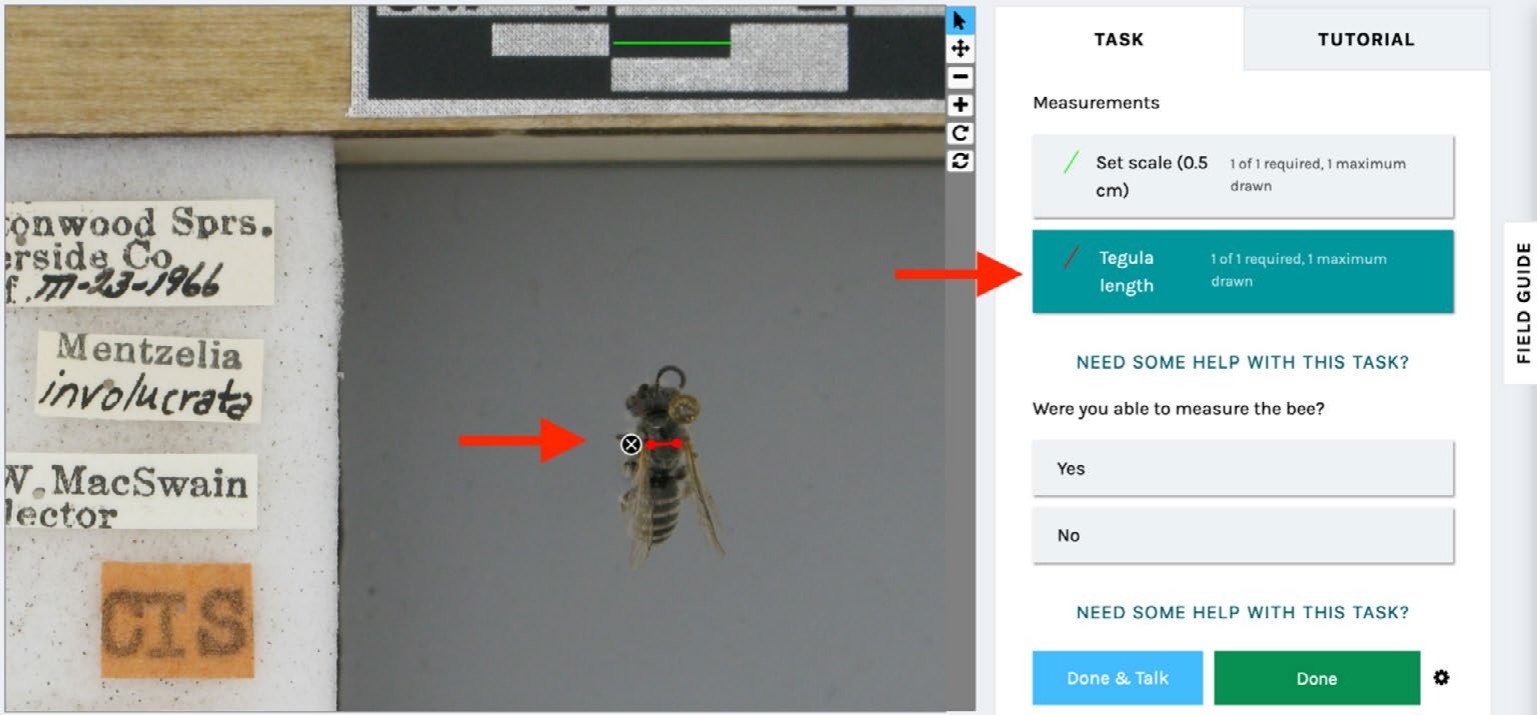
The user interface for bee measurement on the Notes from Nature platform. Users were asked to draw two lines: One spanning a scale bar (in green) and one spanning the intertegular distance (in red; red arrows).

Our Notes from Nature expedition (“Measuring Green Bees”) asked users to help determine the distance between tegulae (sclerites covering the base of the forewing); (Hymenoptera Anatomy Ontology: http://purl.obolibrary.org/obo/HAO_0002584; Yoder et al. 2010; Cane 1987). We instructed Notes from Nature users to first draw a line along a 5 mm length on the ruler visible in the image to calibrate the measurement, then draw a second line connecting the inner margins of the two tegulae (Figure 1). In this way, we obtained coordinate measurements for the inner edges of the tegulae, which we used to calculate ITD. Five unique users independently measured each image. Upon login, users were presented with a short tutorial that described how to measure images (available at https://doi.org/10.5281/zenodo.14927709). Users had the option to seek help from one another and from the research team by posting in an expedition forum. They also were asked to report whether they were unable to accurately measure ITD for each image (typically when the tegula was partially obscured by an antenna). We excluded measurements specified as inaccurate from our analysis. For our analysis, we included only specimens for which five successful measurements were taken, which resulted in a final sample size of 389 specimens, each with five community scientists' measurements. No sensitive user data were collected about participants.

### 2.3 Researcher Measurements

To understand whether community scientist ITD measurements were comparable to research‐grade measurements, we tasked five researchers with measuring ITD from the same sweat bee specimen images (i.e., the 479 Halictinae specimens described above). Researcher measurements were performed in the software ImageJ (Schneider et al. 2012). Each of the five researchers measured every image once, resulting in five measurements per image, to align with our community science dataset. We note that the general task of first setting the scale and then measuring the distance between tegulae is similar for both the citizen science measurement processes and for measurement in ImageJ.

### 2.4 Statistical Analysis

Each specimen image was measured by five unique researchers and five unique community scientists. We calculated the mean, median, and standard deviation of ITD measurements for every image separated by measurement group (researcher vs. community scientist), creating matched pairs of measurements for every specimen image. We then passed these mean, median, and standard deviation values to paired Wilcoxon signed‐rank tests to assess whether researchers and community scientists differ significantly in their measurements. We calculated the effect size as the rank‐biserial correlation coefficient (*r*), which indicates the magnitude of observed differences. We inspected QQ‐plots and determined that data were nonnormal before proceeding with the nonparametric Wilcoxon tests. We calculated the percent error in the mean and median community scientist measurements as follows:

$$\text{Percent error}=\left(\frac{\left|\text{Community scientist}s^{\prime }\mathrm{ITD}-\text{Researcher}s^{\prime }\mathrm{ITD}\right|}{\text{Researcher}s^{\prime }\mathrm{ITD}}\right)\times 100$$

We also calculated the mean signed error (MSE, the average of the signed differences between paired measurements) between community scientist and researcher ITD values, to investigate systematic biases in ITD measurement. Results are reported as mean ± standard error. All analyses were conducted in R version 4.4.2 (R Core Team 2024).

## 3 Results

Fifty‐two community scientists contributed to the Notes from Nature bee measurement project (other users participated without signing in to an account, and so could not be distinguished as unique participants). Each of these users contributed 43.596 ± 12.086 specimen measurements (range: 1–404 measurements). Community scientists reported that they were able to successfully measure ITD for 97.958% of images.

**FIGURE 2:**
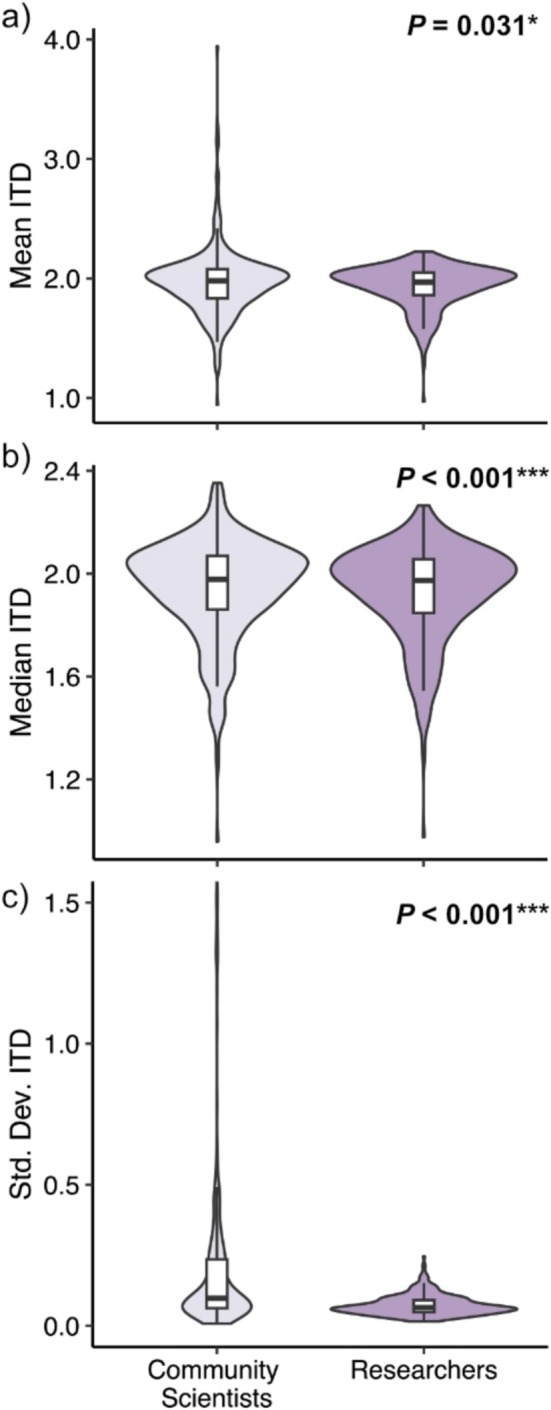
Comparisons between community scientist and researcher measurements of sweat bee intertegular distance (ITD), represented as the (a) mean, (b) median, and (c) standard deviation of the five measurements per specimen conducted by each group (community scientists or researchers). Purple curves indicate the density of measurements, and inlaid white boxplots indicate the median and interquartile range. Community scientists reported significantly larger ITD measurements than did researchers (Wilcoxon tests: Mean ITD: *p* = 0.031; median ITD: *p* < 0.001). Community scientists also reported more variable measurements than did researchers (Wilcoxon test: SD ITD: *p* < 0.001). The *y* axis of plot (c) is truncated at SD = 1.5 to aid readability. Seven outlier values between 1.5 and 3.7 were omitted from the display but included in all statistical analyses.

**FIGURE 3:**
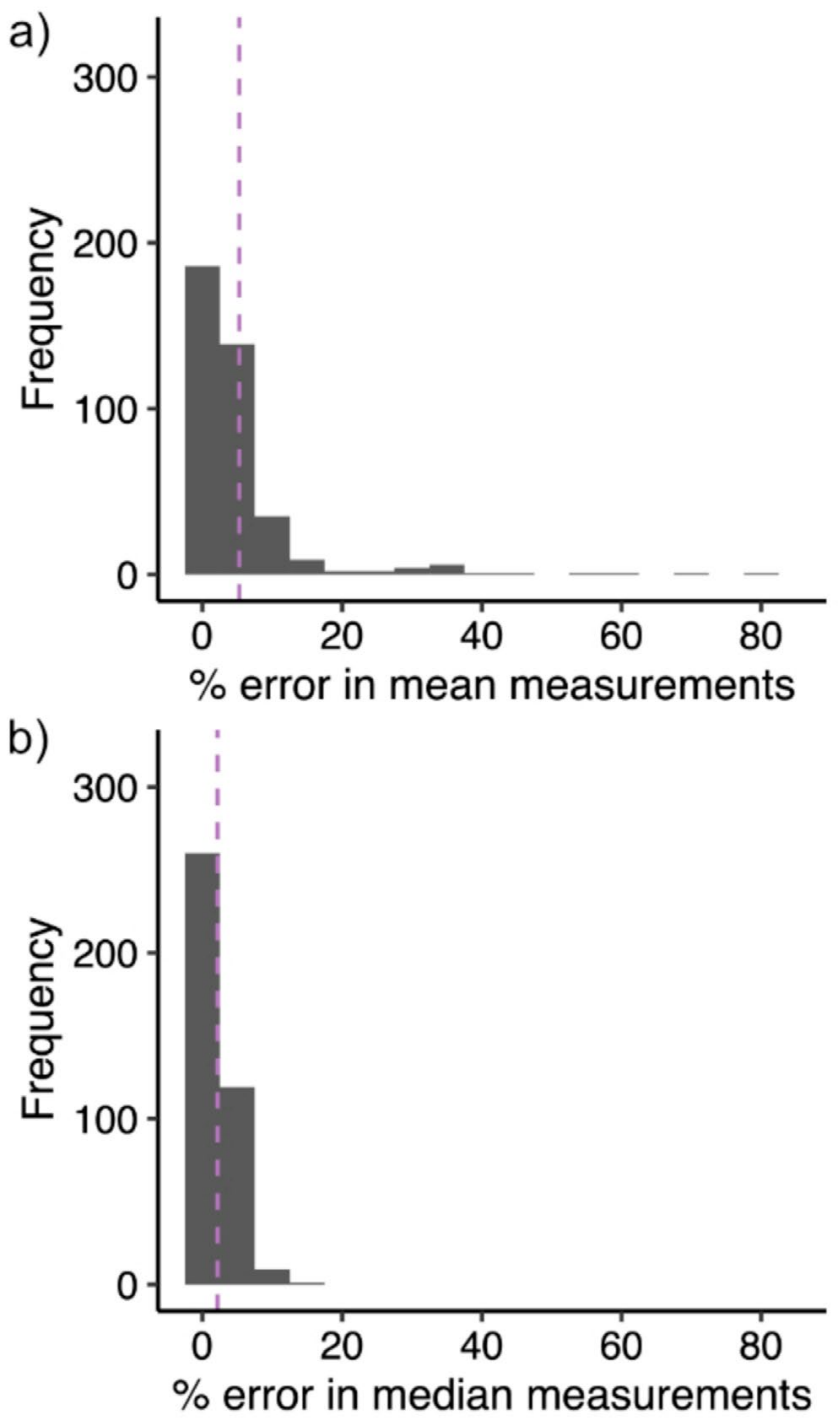
Histograms showing the distribution of values of (a) the percent error in mean and (b) median measurements of ITD when comparing community scientist and researcher measurements. On average, there was a 5.51% error when looking at the mean ITD value for a given specimen, and a 2.27% error when looking at the median ITD value (purple dashed lines).

We obtained ITD measurements for 389 sweat bees, each measured independently by five unique researchers and five unique community scientists. Community scientist measurements of ITD were significantly larger than researcher measurements, both for the mean measurement value of a given specimen (community scientists' mean = 1.958 ± 0.091 mm; researchers' mean = 1.936 ± 0.096 mm; Wilcoxon test: *Z* = −2.454, *p* = 0.031); (Figure 2a) and the median measurement value (community scientists' median = 1.934 ± 0.090 mm; researchers' median = 1.920 ± 0.089 mm; Wilcoxon test: *Z* = −4.679, *p* < 0.001); (Figure 2b). Community scientists also reported significantly more variable measurements than did researchers (community scientists' SD = 0.227 ± 0.011 mm; researchers' SD = 0.072 ± 0.004 mm; Wilcoxon test: *p* < 0.001); (Figure 2c). However, the effect size for the difference in measurements was small (Wilcoxon test: *r*_mean_ = −0.122, *r*_median_ = −0.233). The mean percent error in community scientist measurements was 5.51% when looking at the mean ITD value (Figure 3a) for a given specimen, and a 2.27% when looking at the median ITD value (Figure 3b). Approximately a third of community scientist median measurements (31.620%) had less than 1% error. Compared to researchers, community scientists tended to overestimate ITD by 0.014 mm on average (MSE of median ITD measurements).

## 4 Discussion

The success and recognition of community science research depends on our ability to validate data quality and make appropriate downstream decisions regarding data use (Balázs et al. 2021; Downs et al. 2021; Lukyanenko et al. 2016; Wiggins et al. 2011). We compared community scientist measurements of bee body size to measurements conducted by trained researchers. We found that these measurements differed significantly, but the effect size of this difference was small. These results provide support for community science approaches to some trait measurement tasks, provided that consideration is given to the expected error during analysis.

Our analysis demonstrated that, with only minimal training, community scientists can effectively perform morphological measurement tasks with limited error. Community scientists self‐reported that they were successfully able to perform ITD measurement for 98.0% of images in our sample. Allowing users to report when they were unable to perform measurements accurately was an important quality check in our pipeline, enabling us to remove problematic images from our analysis. Noise is a common feature in crowd‐sourced datasets (Aceves‐Bueno et al. 2017); indeed, community scientists in our study produced more variable measurements than did researchers, which has been reported in other comparisons of citizen scientists with trained researchers (Brenskelle et al. 2020). One likely source of this variability is the difference in the number of participants in each group—more than 50 community scientists contributed measurement data, versus five total researchers. Given the variance inherent to crowd‐sourced data collection, replicating measurements is an important mechanism for improving data quality in community science projects (Kosmala et al. 2016; Wiggins et al. 2011). Taking the median of the five ITD measurement replicates for a given specimen produced more reliable size estimates than the mean, resulting in a 2% error in ITD estimates, or an average overestimate of 0.014 mm. Whether this expected error is acceptable will depend on the research question of interest, and whether a 2% measurement error could obscure relevant differences in body size. For example, studies have documented contemporary bee body size declines at a rate of less than 1% mass or ITD per year (Herrera et al. 2023; Oliveira et al. 2016). Detecting these subtle trends using community scientist measurements may therefore require sufficiently long time series to overcome measurement noise and bias.

In our study, community scientists tended to overestimate ITD relative to researchers. Overestimation in ITD can arise from misidentification of the edges of the tegulae. This measurement error could arise from insufficient training and/or from precision limitations of the measurement software interface used by community scientists on the Zooniverse and Notes from Nature, which differed from that used by researchers (ImageJ). One key difference between the platforms is that the Notes from Nature tool has a larger point selection cursor than ImageJ's, which may obscure the underlying anatomy at the selected points, thus introducing noise when marking the edges of the tegulae. The ImageJ software used by the researcher group in our study allows for more precise point selection at the resolution of individual pixels (Schneider et al. 2012). As designed, our study does not control for these software differences, but rather compares measurements as they would realistically be collected by each group: researchers are unlikely to use Notes from Nature as an in‐lab measurement tool, and ImageJ is not a feasible tool for large‐scale community science projects. As such, we cannot distinguish between error due to software limitations and error due to user differences. Rather, our comparison asks whether measurement projects implemented through Notes from Nature represent high‐quality data collection alternatives to traditional methods. Finally, we selected green halictine bees for our measurement test due to the visibility of the tegulae in these species. Other taxa, especially dark‐colored or hairy bees, may incur greater measurement error due to difficulty identifying the edges of the tegulae and could benefit from additional validation or attention to lighting during the imaging process. Including options for users to self‐report inability to measure particular images is an important safeguard for data quality in these difficult groups.

Improvements to our data collection protocol could increase the suitability of these measurements for research use. In particular, increasing the precision of point selection in the Notes from Nature interface may reduce measurement error. Statistical approaches (e.g., calibration based on researcher measurements) could help account for bias in these datasets by adjusting for known, systematic differences (Bird et al. 2014). Another possibility for reducing error could be to increase the number of measurement replicates per specimen, though the benefits of this approach should be weighed against the time costs for volunteers. Imaging specimens is also a time‐intensive process, and generating images for the purposes of measurement alone would be a greater time investment than simply measuring specimens by hand (e.g., using a microscope reticule). In our case, specimen images were generated as part of workflows focused on eventual label transcription, then reused for ITD measurement. These dual uses of image data highlight the value of including specimens and relevant references (i.e., rulers or color standards) in digitization pipelines.

Trait measurement represents an exciting avenue for public participation in biodiversity research. Our participants were persistent and engaged in the size measurement task, with each community scientist measuring more than 40 specimens on average. Compared to label transcription tasks, measurement tasks could offer more active means of engagement with museum specimens and with hypothesis‐driven research. These measurements could be leveraged to answer pressing evolutionary and conservation questions about interspecific body size variation in bees, for example, supporting studies of pollination ecology (Cullen et al. 2021), movement ecology (Greenleaf et al. 2007; Kendall et al. 2022), or community responses to environmental change (Bartomeus et al. 2013; Benjamin et al. 2014; Kammerer et al. 2021). These methods may be even better suited to larger‐bodied organisms (e.g., vertebrates), for which small measurement errors represent a smaller proportion of total body size. Broadly, these results highlight the potential of community science approaches for expanding our understanding of morphological and functional change across taxa using natural history collections.

## Author Contributions

**Madeleine M. Ostwald:** conceptualization (equal), data curation (equal), formal analysis (lead), writing – original draft (lead), writing – review and editing (lead). **Colleen Smith:** conceptualization (supporting), data curation (equal), formal analysis (supporting), writing – review and editing (equal). **Julie Allen:** conceptualization (equal), writing – review and editing (supporting). **Alec Buetow:** data curation (equal), formal analysis (supporting), investigation (equal), writing – review and editing (supporting). **A. Rosie Manner:** methodology (equal), writing – review and editing (supporting). **Robert Guralnick:** conceptualization (equal), writing – review and editing (equal). **Carys Goldsmith:** data curation (equal), writing – review and editing (supporting). **Katja C. Seltmann:** conceptualization (lead), funding acquisition (lead), writing – review and editing (equal).

## Conflicts of Interest

The authors declare no conflicts of interest.

## Data Availability

All data associated with this manuscript is available at https://doi.org/10.5281/zenodo.14927709.
